# Analysis of nucleosome positioning landscapes enables gene discovery in the human malaria parasite *Plasmodium falciparum*

**DOI:** 10.1186/s12864-015-2214-9

**Published:** 2015-11-25

**Authors:** Xueqing Maggie Lu, Evelien M. Bunnik, Neeti Pokhriyal, Sara Nasseri, Stefano Lonardi, Karine G. Le Roch

**Affiliations:** Department of Cell Biology and Neuroscience, Institute for Integrative Genome Biology, Center for Disease Vector Research, University of California, Riverside, 900 University Avenue, Riverside, CA 92521 USA; Department of Computer Science and Engineering, University of California, Riverside, 900 University Avenue, Riverside, CA 92521 USA

**Keywords:** Malaria, Nucleosome, Gene prediction, Transcription, Non-coding RNA, Genome annotation

## Abstract

**Background:**

*Plasmodium falciparum,* the deadliest malaria-causing parasite, has an extremely AT-rich (80.7 %) genome. Because of high AT-content, sequence-based annotation of genes and functional elements remains challenging. In order to better understand the regulatory network controlling gene expression in the parasite, a more complete genome annotation as well as analysis tools adapted for AT-rich genomes are needed. Recent studies on genome-wide nucleosome positioning in eukaryotes have shown that nucleosome landscapes exhibit regular characteristic patterns at the 5’- and 3’-end of protein and non-protein coding genes. In addition, nucleosome depleted regions can be found near transcription start sites. These unique nucleosome landscape patterns may be exploited for the identification of novel genes. In this paper, we propose a computational approach to discover novel putative genes based exclusively on nucleosome positioning data in the AT-rich genome of *P. falciparum*.

**Results:**

Using binary classifiers trained on nucleosome landscapes at the gene boundaries from two independent nucleosome positioning data sets, we were able to detect a total of 231 regions containing putative genes in the genome of *Plasmodium falciparum*, of which 67 highly confident genes were found in both data sets. Eighty-eight of these 231 newly predicted genes exhibited transcription signal in RNA-Seq data, indicative of active transcription. In addition, 20 out of 21 selected gene candidates were further validated by RT-PCR, and 28 out of the 231 genes showed significant matches using BLASTN against an expressed sequence tag (EST) database. Furthermore, 108 (47 %) out of the 231 putative novel genes overlapped with previously identified but unannotated long non-coding RNAs. Collectively, these results provide experimental validation for 163 predicted genes (70.6 %). Finally, 73 out of 231 genes were found to be potentially translated based on their signal in polysome-associated RNA-Seq representing transcripts that are actively being translated.

**Conclusion:**

Our results clearly indicate that nucleosome positioning data contains sufficient information for novel gene discovery. As distinct nucleosome landscapes around genes are found in many other eukaryotic organisms, this methodology could be used to characterize the transcriptome of any organism, especially when coupled with other DNA-based gene finding and experimental methods (e.g., RNA-Seq).

**Electronic supplementary material:**

The online version of this article (doi:10.1186/s12864-015-2214-9) contains supplementary material, which is available to authorized users.

## Background

As one of the world’s most deadly infectious diseases, malaria is responsible for about 584,000 deaths annually, the vast majority of which are children under the age of five [1]. Currently, no approved vaccine is available for disease prevention, and the rapid development of parasite resistance to current antimalarial drugs is a major challenge for the control of malaria. Out of five human malaria parasite species, *Plasmodium falciparum* causes 90 % of all malarial deaths [1]. *P. falciparum* has a complex life cycle involving multiple stages in two host organisms, humans and mosquitoes. This multi-stage life cycle is tightly regulated, presumably by strict control of stage-specific gene expression. However, the mechanisms regulating gene expression in *P. falciparum* are still poorly understood. In particular, relatively few specific transcription factors and regulatory elements have been identified [2, 3]. In addition, the annotation of protein coding and non-protein coding genes is incomplete. To facilitate our understanding of the parasite’s life cycle and its regulatory mechanisms and thus assist the development of antimalarial drugs, a more accurately annotated genome is needed.

The draft of the annotated genome of *P. falciparum* was first published in 2002 [4]. *P. falciparum* has a relatively compact genome consisting of fourteen chromosomes with a total length of approximately 23 Mb [4]. The *P. falciparum* genome is the most AT-rich eukaryotic genome sequenced to date, with an overall AT-composition of 80.7 %, rising to 90-95 % in introns and intergenic regions [5]. Currently, 5,777 predicted protein coding genes have been reported (plasmoDB v26) and ~50 % of these genes share little or no sequence similarity to genes or the encoded proteins in other organisms [4–6]. The average gene length in *P. falciparum* is 2.3 kb and the average length of intergenic regions is ~1.7 kb [7]. Both computational and evidence-based gene-finding methods have been applied to obtain gene annotations. Genome annotations of the reference strain 3D7 were performed *in silico* using software tools including Artemis, Genefinder, GlimmerM, and phat [8, 9]. Most of the predicted genes have been verified using various experimental techniques including full-length cDNA, expressed sequence tag (EST), and mass spectrometry analysis, among others [7, 10–13]. More comprehensive annotations of the parasite’s gene structure and other functional elements have been possible since the advent of second-generation sequencing technology [6, 13–18].

Despite significant advances in the analysis of the parasite’s genome, genome annotation in *P. falciparum* is still a work in progress. The AT-richness and the relative lack of sequence homology to other organisms hamper the application of sequence-based gene prediction tools and complicate the identification of functional DNA elements, such as protein-binding sites, promoters, or TATA-like boxes. In addition, as mentioned earlier, the parasite has a complicated multi-stage life cycle involving multiple hosts. Due to technical challenges, it is nearly impossible to capture the transcriptome at all different life cycle stages. We are therefore still in need of an improved genome annotation, as well as analysis tools capable of handling the parasite’s AT-rich genome that will help us to better understand the regulatory mechanisms controlling gene expression in the parasite.

In mammalian genomes, a large number of non-coding transcripts have been identified based on chromatin signatures H3K4me3 and H3K36me3 [19]. This finding suggests that elements defining and bracing chromatin architecture may be used to assist the identification of undiscovered genes. In this study, we present a machine learning approach to predict genes in *P. falciparum* that is completely independent from the primary DNA sequence, but instead exploits the underlying chromatin structure and nucleosome landscape. The fundamental unit of chromatin is a nucleosome, a stretch of ~147 bp of DNA wrapped around a core of eight histone proteins. Nucleosomes are distributed non-uniformly around genes, and this distinct nucleosome landscape is known to play an important role in gene regulation. In particular, the core promoter is usually characterized by a nucleosome-depleted region that allows the binding of transcription factors and facilitates the assembly of the transcription preinitiation complex [20, 21]. Previous studies in our lab have highlighted several common and unique eukaryotic features of the *P. falciparum* nucleosome landscape. Similar to other eukaryotes, *Plasmodium*’s promoters and transcription start sites are relatively nucleosome depleted, and nucleosome occupancy is higher inside genes as compared to intergenic regions [22–24]. However, in contrast to the strongly positioned +1 nucleosome directly downstream of the transcription start site in other eukaryotes [20, 25–28], the most strongly positioned nucleosomes in *P. falciparum* are located at the start and end of the open reading frame [22, 23]. Based on these nucleosome landscape characteristics, we propose a novel method for gene detection using classifiers trained on nucleosome profiles of annotated genes. Other experimental methods used for gene detection, such as RNA-Seq or expressed sequence tags (EST), can be noisy, potentially resulting in false predictions. Therefore, our methodology may serve as a complementary approach for refining genome annotations, especially coupled with sequence-based gene predictions and other experimental methods.

## Results

### Building a classifier on nucleosome positioning profiles

**Fig. 1:**
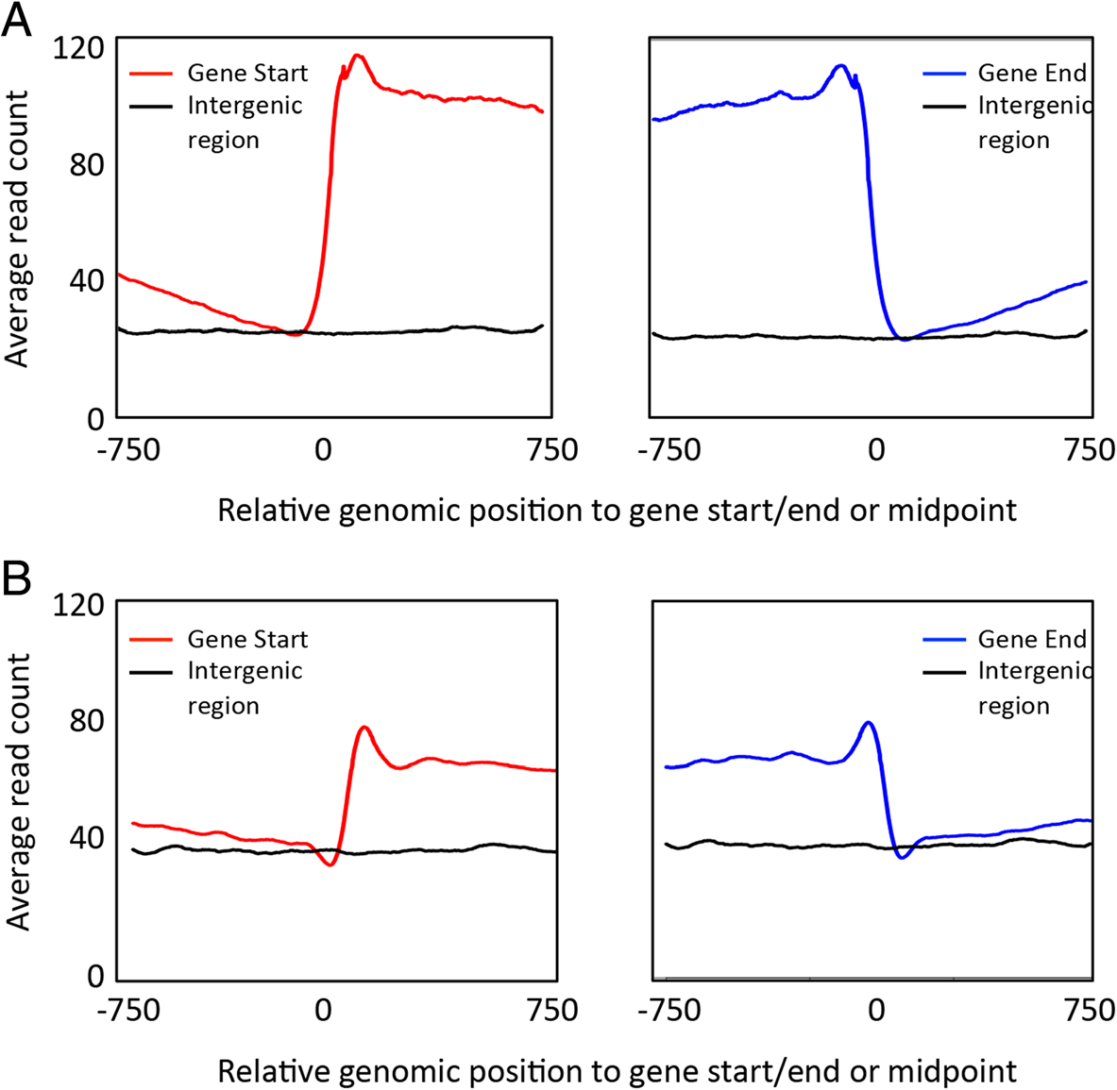
Nucleosome occupancy patterns in *P. falciparum*. **a** Average sequence coverage profiles around the start (left panel) and the end (right panel) of genes (colored line), and in intergenic regions (black line) in the nucleosome occupancy data set from Bunnik et al. [22] (data set B1). **b** Similar analysis for the nucleosome occupancy data set from Bartfai et al. [30] (data set B2). In all windows, the genomic position indicated on the x-axis is relative to the location of the gene start/end, or to the midpoint of intergenic windows

In a previous study, our lab has used second-generation sequencing to generate high-resolution nucleosome positioning profiles for three different stages of *P. falciparum*’s asexual cycle [22]. This data set revealed a distinct nucleosome landscape around genes, with higher nucleosome occupancy inside genes, lower nucleosome coverage in intergenic regions, and strongly positioned nucleosomes at the gene boundaries (Fig. 1a). In addition, as observed in other eukaryotic genomes [26, 27, 29], a nucleosome-depleted region was found immediately upstream of the transcription start site, which likely harbors the binding sites of transcription factors [22]. These observations were replicated using an independently generated *P. falciparum* nucleosome occupancy data set [22, 30] (Fig. 1b). In this paper, we exploited this nucleosome landscape around genes to identify regions in the genome containing putative novel genes. To gain additional power for gene detection, we decided to predict the presence of novel genes using the two independently published nucleosome positioning data sets [22, 30]. For each data set, we summed the sequence coverage profile at each of the parasite’s asexual stages into a single genome-wide nucleosome positioning data set. This resulted in a total of two combined profiles, namely i) profile B1 from Bunnik et al. [22] consisting of three asexual cycle time points, and ii) profile B2 from Bartfai et al. [30] consisting of four asexual cycle time points.

From each of the combined nucleosome profiles, we extracted windows that either contained a gene start within its defined central region (positive class windows) or were completely derived from intergenic regions (negative class windows) (see Methods; Additional file 1: Figure S1). We then used these positive and negative class windows to train a binary classifier (i.e., a support vector machine with RBF kernel) to recognize the general nucleosome occupancy pattern at gene start codons. The parameters of the classifier were optimized using cross validation (see Methods; Additional file 1: Figure S2). In parallel, an independent classifier was trained on the nucleosome landscape at gene stop codons. Since we observed that nucleosome landscapes on the forward and reverse strands have slightly different characteristics, we independently optimized both strand-specific and non-strand-specific classifiers. All classifiers performed in very similar ways and optimized classifiers from both data sets gave total recall rates between 91 and 95 % (Additional file 1: Table S1).

**Fig. 2:**
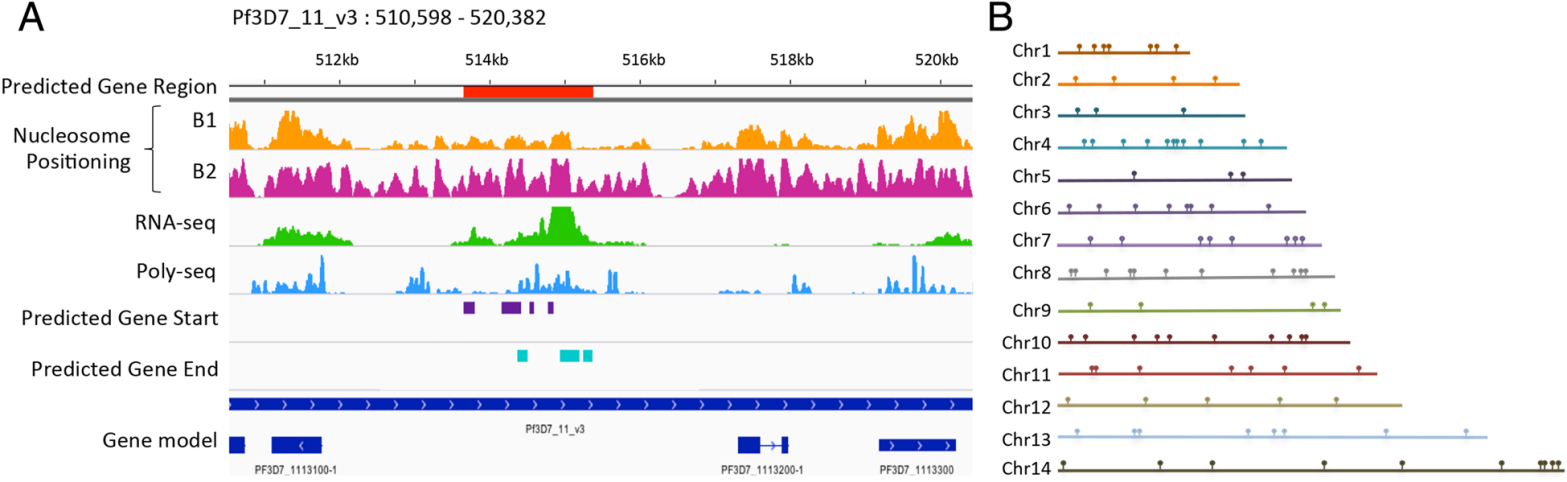
Characterization of regions containing putative novel genes. **a** Genome browser view of an intergenic region containing a predicted gene region (Pf3D7_11_v3: 513,659 – 515,381, shown in red). Predicted gene starts and gene ends are indicated in purple and teal, respectively. This putative novel gene shows sequence coverage in both steady-state RNA-seq (green) and polysomal RNA-seq (blue) data sets. **b** Random distribution of 97 regions predicted using classifiers trained on data set B1 across the 14 chromosomes of the *P. falciparum* genome

These classifiers were then used on the nucleosome landscape of the whole *P. falciparum* genome to detect putative novel gene starts and ends. A sliding-window method was used to scan intergenic regions for the presence of predicted gene starts or gene ends. The classifier produced a confidence score between 0 and 1 for each prediction. A valid gene candidate was defined as a locus with a gene start and a gene end predicted using the same strand classifier with confidence scores above 0.7 and located within the same intergenic region (Fig. 2a). No additional constraint on the distance between a predicted gene start and gene end was required, given the relatively short length of intergenic regions in the genome of *P. falciparum* (1.7 kb on average). A total of 298 final candidate regions with an average segment length of 1 kb were manually identified, of which 97 were detected using the B1 nucleosome positioning profile, and 201 were identified using the B2 nucleosome positioning profile (Additional file 2). Of the 298 candidate regions, 67 genes were identified in both B1 and B2 data sets with an average overlap in predicted gene region of 81 %. This intersect between genes predicted by both data sets was highly statistically significant (*P* < 7.422e-66, calculated based on an hypergeometric distribution analysis [31]). Since overlapping regions may represent alternative splicing variants of the same gene, we merged overlapping regions using mergeBed (BEDtools [32]), resulting in a total of 231 unique regions harboring potential novel genes. All putative novel genes are uniformly distributed over the 14 chromosomes of the *P. falciparum* genome (Fig. 2b).

**Fig. 3:**
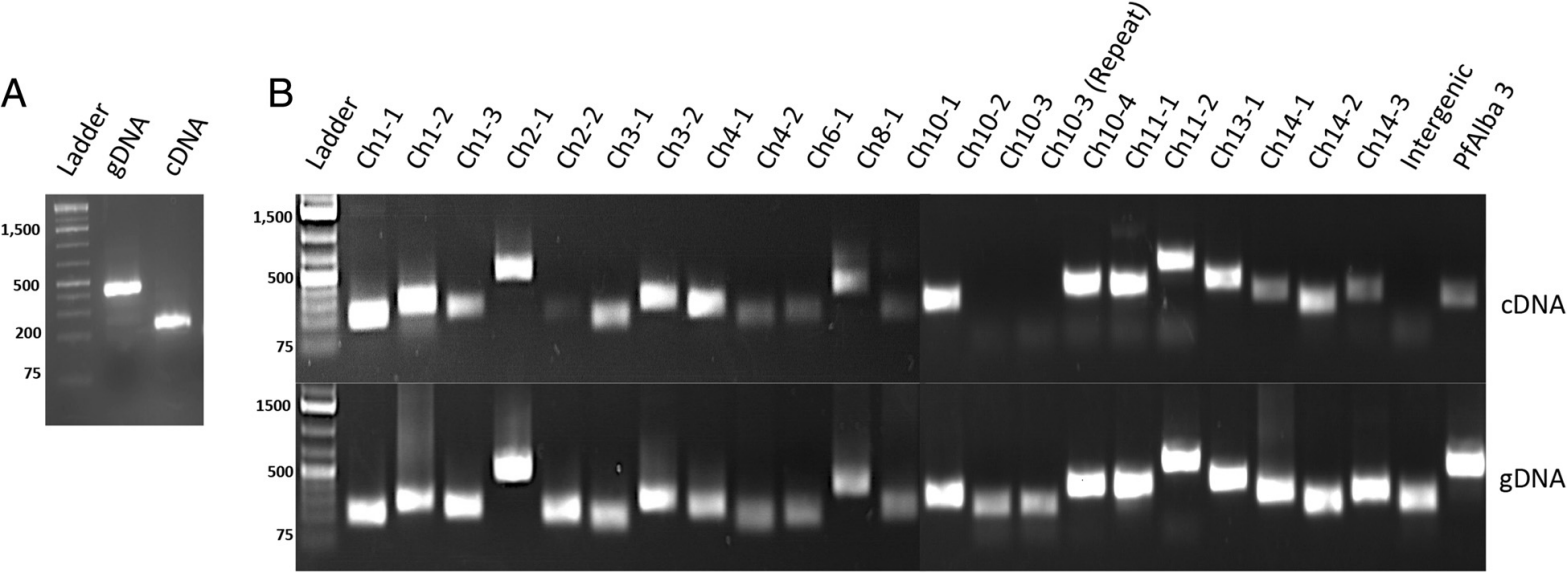
RT-PCR validation of 21 predicted novel genes. **a** Amplification of a fragment of PfAlba3 (PF3D7_1006200) using genomic DNA (middle lane) or cDNA prepared from DNase-treated total RNA (right lane) as a template. Primers were designed on both sides of intron 1, yielding a 429 bp PCR product from genomic DNA and a 164 bp PCR product from cDNA. The presence of a single 164 bp PCR product amplified from cDNA confirms the absence of gDNA contamination. **b** Out of our 231 novel candidate genes, we chose 21 regions for validation using reverse transcription polymerase chain reaction (RT-PCR). The top panel shows amplification products using DNase-treated cDNA as a template, while the bottom panel shows the control reactions using genomic DNA as a template. Of the 21 gene tested, we were able to amplify 20 of the predicted regions. As a control, we were unable to amplify a fragment of intergenic region that was not predicted to contain any genes (marked as “intergenic”)

Among these 231 predicted genes, 88 showed a signal (defined as an average of two or more reads per base) in a previously obtained RNA-Seq data set [18], which we considered strong evidence for the presence of a transcribed gene in this region (Additional file 2). On average, predicted gene regions are covered by eight reads per base, which is significantly higher than that the RNA-Seq coverage in intergenic regions of the same length (Table 1, *P* = 0.015, bootstrap Welch *t*-test with *n* = 100,000). In addition, 108 out of these 231 (47 %) uniquely predicted regions overlap with previously identified long non-coding RNAs (lncRNAs), defined as non-coding transcripts larger than 200 bp that are not antisense or circular RNA [17, 33–36]. To further confirm transcriptional activity in the predicted gene regions, we designed a set of primers targeting 21 selected candidate regions. We were able to amplify 20 of the 21 targeted fragments from cDNA (Fig. 3 and Additional file 3), suggesting that the majority of candidate genes may indeed be transcribed.

**Table 1:** Characteristics of the 231 putative novel genes in comparison with annotated *P. falciparum* genes

	Average RNA-seq coverage	Average Poly-seq coverage	Average GC%	Average length (bp)	Avgerage Nuc coverage (B1)	Average Nuc coverage (B2)	Average H2A.z coverage	Average H3K36me3 coverage	Average H3K4me3 coverage	*n*
Exon	58	30	27	949	35	77	20	41	84	14,795
Gene	75	34	23	2,494	37	69	17	50	83	5,680
Intergenic^a^	3	7	13	1,000	8	37	31	10	22	1,565
Published lncRNA [17, 33–36]	10	7	15	1,114	12	47	35	16	37	986
Predicted genes (B1)	13	4	18	934	22	62	29	25	63	97
Predicted genes (B2)	9	5	16	1,010	17	62	35	18	49	201
All predicted genes (B1 + B2)	8	4	16	1,004	17	62	34	19	50	231

^a^Intergenic regions were defined as the middle 1 kb of all non-coding regions longer than 1,500 bp that do not overlap with annotated genes, predicted genes, or previously identified lncRNAs

### Characteristics of candidate novel *P. falciparum* genes

**Fig. 4:**
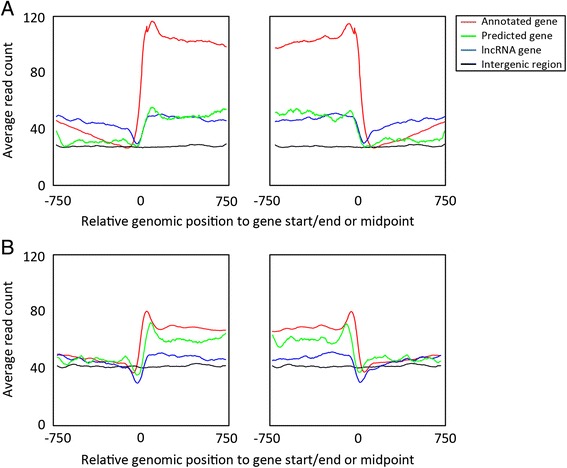
Nucleosome positioning profile around predicted genes. **a** Average sequence coverage profiles around the start (left panel) and the end of genes (right panel) for annotated protein-coding genes (red), published lncRNA genes (blue) and predicted novel genes identified in this study (green) in the nucleosome occupancy data set from Bunnik et al. [22] (data set B1). The average nucleosome occupancy in intergenic regions is presented as a reference (black). **b** Similar analysis for the nucleosome occupancy data set from Bartfai et al. [30] (data set B2). In all windows, the genomic position indicated on the x-axis is relative to the location of the gene start/end, or to the midpoint of intergenic windows

To further investigate the putative genes identified in this study, we compared several characteristics of the predicted regions with known coding and non-coding regions in the *P. falciparum* genome. The average length of the predicted gene is 1,004 bp, which is similar to the average length of exons and lncRNAs in *P. falciparum*. The average GC-percentage for the predicted genes (16 %) is lower than known coding genes (23 %), but close to previously identified lncRNA regions (15 %) and slightly higher than intergenic regions (13 %) (Table 1 and Additional file 1: Figure S3A). Similarly, the average nucleosome occupancy in predicted gene regions ranged between that of known protein-coding genes and that of lncRNA genes (Additional file 1: Figure S3B-C). The nucleosome profiles at the predicted gene starts and gene ends recapitulate the nucleosome features observed in annotated genes, albeit at lower average nucleosome levels (Fig. 4). Furthermore, the predicted novel genes have similar expression levels in steady-state mRNA-Seq [18] and polysome-associated mRNA-Seq [18] data sets as compared to lncRNA genes (Additional file 1: Figure S3D-E). Lastly, we examined the patterns of histone variant H2A.Z and histone marks H3K4me3 and H3K36me3. In *P. falciparum,* H2A.Z is almost exclusively found in nucleosomes located in intergenic regions [30], while H3K4me3 is enriched at the gene boundaries and H3K36me3 is enriched inside gene bodies [37] (Additional file 1: Figure S4). We found that the average H2A.Z occupancy is higher in predicted genes than in annotated genes, and very similar to intergenic and previously identified lncRNA genes (Table 1 and Additional file 1: Figure S3F). In line with H3K36me3 being more abundant in coding regions as compared to noncoding regions in *P. falciparum*, we observed that the abundance of H3K36me3 in our predicted genes is in between that of coding and non-coding regions*.* In addition, H3K36me3 levels in our predicted genes are higher than in previously identified lncRNAs (Table 1 and Additional file 1: Figure S3G). Similar to the H3K36me3, H3K4me3 occupancy in our predicted genes is also found to be higher than in previous identified lncRNA and ranged between coding and non-coding regions (Table 1 and Additional file 1: Figure S3H).

As the majority of the predicted genes showed characteristics similar to those of lncRNA genes, we further classified our novel gene candidates into putatively protein-coding and non-protein-coding genes using a previously generated polysome-associated mRNA-Seq data set [18], which provides a snapshot of transcripts that are actively being translated. Using a cutoff of an average sequence depth of two reads across the entire predicted gene region, 73 out of 231 putative novel genes were found to be associated with polysomes and are thus potentially translated. For each predicted gene region, the longest open reading frame (ORF) was identified using ORF Finder with default values [38]. More putatively protein-coding gene candidates (6 out of 73 [8.2 %]) than putatively non-coding gene candidates (3 out of 158 [0.2 %]) contain an ORF longer than 100 amino acids (two-tailed Fisher’s exact test, *P* = 0.03). In addition, putatively coding regions tend to have larger ORFs (average of 55 aa) than putatively non-coding regions (average of 48 aa, two-tailed Student’s *t*-test, *P* = 0.07). On the other hand, the fraction of regions that does not contain an ORF larger than 30 amino acids is similar between both groups of gene candidates: 10 out of 73 (13.7 %) of putative protein-coding regions versus 21 out of 158 (13.3 %) of putative non-protein-coding regions (Additional file 2). However, 135 out of 231 novel genes have multiple non-overlapping ORFs on the same strand that could be exons belonging to a single gene. We could not find any evidence for splicing events in the RNA-seq data, although it should be mentioned that the sequence coverage in these regions is relatively low and may not allow the detection of such events.

### Homology search

Comparative genomics is a powerful approach to gather evidence about putative genes. To find homologs of our putative novel genes, we aligned the predicted regions with known protein transcripts from the Uniprot-Trembl database using BLASTX [39–41]. Using stringent search settings (perfect matched length > 30 % and e-value < 1e-5), no significant hits were found, suggesting that all of our predicted genes may be parasite-specific. This result is expected, since more than 50 % of *P. falciparum* genes are unique to the parasite and majority of the putative genes identified in this study may be non-coding genes with low sequence conservation. Next, we searched against the reference RNA sequences (refseq_rna) database using the discontiguous MegaBLAST program of BLASTN that is tailored to more dissimilar sequences [40–42]. A similarity cutoff of e-value < 1e-5 resulted in two significant matches. One of the predicted gene regions (Pf3D7_14_v3:38,574-40,547) showed ~50 % query coverage and more than 70 % identity with approximately twenty of the *var* genes, while another putative gene region (Pf3D7_08_v3:1,288,505-1,289,391) showed more than 40 % query coverage and 70 % identity with ribosomal RNA sequences across protozoan species, including *Plasmodium vinckei vinckei*, *Theileria orientalis*, and *Babesia equi*. We also used BLAST to compare the candidate regions with a database of known expressed sequence tags (EST) and found 28 matches, the majority of which are derived from *P. falciparum* (Additional file 2). These findings provide independent evidence that our predicted regions might indeed contain novel genes.

## Discussion

In this paper, we have used a machine learning approach for the detection of genes in the AT-rich genome of the human malaria parasite, *P. falciparum*, using exclusively nucleosome positioning data. Using classifiers trained on two independent nucleosome occupancy data sets, we detected a total of 231 putative novel genes. Eighty-eight of these 231 newly predicted genes exhibited transcription signal in RNA-Seq data and twenty out of 21 putative gene regions were validated by RT-PCR, indicating that our methodology is highly successful in identifying genes. Furthermore, of all putative gene regions identified using the nucleosome occupancy data set from Bunnik et al. [22], 69 % were confirmed in the nucleosome positioning data set from Bartfai et al. [30], indicating that the classifiers trained on these two independently generated nucleosome landscapes are in good agreement. Collectively, our results demonstrate that local chromatin structure is sufficiently informative for genome annotation. Gene predictions based on nucleosome positioning datasets could thus be used to complement and augment sequence-based methodologies that are currently used for this purpose.

Based on the evidence we collected, it seems likely that many of the regions predicted here encode long non-coding RNAs. First, 108 of the predicted regions have been previously identified as lncRNA genes [17, 33–36]. Second, the sequence (GC-content) and nucleosome occupancy characteristics of the predicted regions are more similar to known lncRNAs than to protein-coding genes. Third, few of the predicted regions contain large ORFs. In other eukaryotic organisms, lncRNAs have been shown to be involved in the regulation of a multitude of cellular processes, one of which is regulation of gene expression by targeting general transcription factors and inducing chromatin remodeling [43–48]. In *P. falciparum*, identification and functional characterization of lncRNAs is ongoing. Most studies have focused on the identification of long non-coding telomeric end-associated transcripts that are similar to telomeric repeat-containing lncRNAs (TERRA) found in human and that are important for telomere maintenance [13, 34, 49]. Some of these lncRNAs contain binding sites for PfSIP2, a transcription factor specific to *Plasmodium* that is thought to be involved in regulation of *var* genes [34, 50]. This gene family is responsible for pathogenesis and immune evasion and most of its members are located in subtelomeric regions. These lncRNAs are likely to play important regulatory roles in *var* gene silencing by inducing heterochromatin formation, thus creating a repressive environment at the telomeric and subtelomeric ends [13, 34, 49, 51]. Additionally, lncRNAs have been implicated in various other processes, such as metabolic, biosynthetic and regulatory activities [13, 43, 52–55]. Our experimental results have expanded the list of putative lncRNAs in *P. falciparum*, and it will be of great interest to further validate and characterize these transcripts to understand their function in parasite biology.

Unfortunately, we were unable to use nucleosome positioning as a means to discover novel genes in the telomeric regions. Due to aberrant nucleosome positioning in the telomeric and centromeric regions compared to the rest of the genome, we had to exclude these regions from our gene predictions. The number of known lncRNAs derived from these regions is too small (*n* = 22) for accurate training of a separate classifier on these atypical parts of the genome.

In addition to putative lncRNAs, we also distinguished 73 regions that may contain protein-coding genes, based on the association of their transcripts with polysomes. The polysome profiling data set used in this study was obtained by separating polysomes on a sucrose gradient, followed by isolation and sequencing of mRNA in the polysome fractions [18]. This methodology provides a catalogue of transcripts that are actively being translated. However, it also captures polyadenylated transcripts that are merely associated with polysomes as regulatory elements, or that are present in ribonucleoprotein complexes that co-sediment with polysomes. Based on polysome profiling data alone, it is therefore impossible to determine whether a gene encodes a protein. Further study will be necessary to determine the translational status of the putative protein-coding genes identified in this study.

Beside protein-coding genes and genes encoding lncRNAs, a third option for regions identified in this study is to contain pseudogenes. For decades, pseudogenes have been considered non-functional or ‘junk’ DNA; however, the conserved sequence similarity between pseudogenes and coding genes suggests a selective maintenance of these non-coding elements. They may have an important biological role that has not yet been fully understood. In recent mammalian studies, transcripts of pseudogenes showed regulatory roles, largely through antisense mechanisms [56, 57]. Expressed pseudogenes have also been implicated in mRNA stability in transgene mouse mutants [58]. Similar regulatory pseudogenes may also be present in *P. falciparum*, in particular in predicted gene regions with homology to annotated genes as identified using BLAST searches.

As a selection criterion for the identification of regions containing putative novel genes, we used the presence of both a gene start and a gene end within the same intergenic region. However, we also identified regions with only a predicted gene start or a gene end, but not both. Often, the intergenic regions containing these single-end predictions do show sequence coverage in the steady state or polysomal RNA-Seq data sets. Possible explanations for such single-end predictions include the presence of genes coding for small transcripts that are difficult to capture using a nucleosome positioning dataset. Each nucleosome covers approximately 146 base pairs of DNA, raising the possibility that short genes do not show distinct nucleosome occupancy features. Alternatively, the nucleosome features at the other end of the predicted gene region may be irregular and therefore not meet the quality threshold for selection.

## Conclusion

In this study, we have demonstrated that using a machine learning approach trained on the nucleosome landscape around genes, we were able to identify 231 putative genes, of which the majority showed evidence of expression in RT-PCR, EST, steady-state RNA-Seq, or polysomal RNA-Seq data sets in the malaria parasite, *P. falciparum*. A similar methodology could be used for predicting the location of transcription start sites (TSSs), since TSSs are generally marked by an upstream nucleosome-depleted region. Therefore, this approach may ultimately be useful to identify key regulatory elements and to complement other sequence-based genome annotation efforts, which will provide further insights into gene regulatory mechanisms in *P. falciparum*. Furthermore, similar machine learning approaches may also be applied to other organisms as long as a nucleosome-positioning data set is available and the nucleosome landscape around genes shows regular periodic characteristics.

## Methods

### Nucleosome positioning profiles

Nucleosome positioning profiles of the three main stages of *P. falciparum*’s asexual replication cycle were generated by micrococcal nuclease digestion of formaldehyde-crosslinked chromatin followed by chromatin immunoprecipitation using an antibody against histone H3. Nucleosome-bound DNA fragments were sequenced on the Illumina HiSeq platform as described in [22, 30]. Two *P. falciparum* 3D7 nucleosome positioning profiles were used in this study. Data set B1 from Bunnik et al. [22] consists of three asexual cycle time points (SRP026365), while data set B2 from Bartfai et al. [30] consists of four asexual cycle time points (SRP003508). Reads were trimmed, mapped to *P. falciparum* 3D7 genome version 9.0 and were converted into coverage profiles by counting the number of sequence reads mapped at each nucleotide position as described in [22]. For each dataset, all coverage profiles were summed to generate a combined nucleosome profile *G* to be used as input data to train the classifier. The telomere and centromere regions display aberrant nucleosome coverage compared to the rest of the genome and were therefore removed from this data set.

By sliding a window of length *w* along the combined genome-wide profile *G* with a sliding step of *h* = 1 base pairs, we converted the input *G* into a set *D* of windows. Each window in *D* is a vector of length *w*, and each coordinate *i* of the vector represents the total number of mapped reads at location *i*. Inside each window, we defined a central region of length *m*, called *margin*. The total number of windows *n* is $\left\lceil \frac{\left(\left|G\right|-w+1\right)}{h}\right\rceil$, the coordinates of a window *D*_*i*_ (*i* = 1, 2, 3 … *n*) is [*a*_*i*_*, b*_*i*_] = [(*i* − 1)*h* + 1, (*i* − 1)*h* + *w*] and the coordinates of the margin window *D*_*i*_ is $\left[ ai+\frac{w}{2} - \frac{m}{2},\  ai+\frac{w}{2} + \frac{m}{2}\right]$.

After extracting the windows, we assigned a label to each window depending on the presence or absence of a gene start or end, as defined below. Only the positive class and negative class windows were used to train the binary classifier for gene recognition. We defined a *negative class* as a window that does not overlap with any gene (intergenic windows), a *positive class* as a window that contains a gene start (or gene end for the detection of gene ends) inside the margin, and *other class* as a window that does not fall into the categories of positive or negative windows.

### Cross validation

The following section refers to the detection of gene starts. For gene ends, we used the same approach. To differentiate gene start sites and intergenic regions, a binary classifier was trained on positive class windows and negative class windows. Two randomly sampled data sets of windows were used interchangeably as training set or test set. One was sampled from windows of odd chromosomes, while the other was sampled from windows of even chromosomes. For each choice of parameter, we ran ten experiments. Odd chromosome windows were used as training and even chromosome windows were used for testing in the first five experiments, and vice versa for the other five experiments. All data was normalized with zero mean and unit variance. To evaluate the classifier’s performance, we computed accuracy, precision and recall as described below.

$$ Accuracy=\frac{TP\ }{TP+FN} $$

$$ Precision=\frac{TP}{TP+FP}\kern0.75em \left( = \mathrm{specificity}\right) $$

$$ Recall = \frac{TP}{TP+FN}\kern0.5em \left( = \mathrm{sensitivity}\right) $$

$$ F- score=\frac{2 \times Precision \times Recall\ }{Precision+ Recall} $$

Recall and precision often show an inverse relationship, where it is possible to increase one at the cost of reducing the other. For our purpose of finding putative genes, the primary goal was to obtain the highest possible recall for both positive and negative classes.

### Support vector machine classifier

Support vector machine (SVM) is a family of binary classifiers than can learn from a training set to discriminate between positive and negative examples by finding a hyperplane that maximizes the margin [59]. To choose the best kernel for the SVM, we first used principal component analysis (PCA) to explore the relationship between the positive and negative classes, and then investigated different SVM kernels available from the Scikit-learn packages [60, 61]. Based on cross-validation experiments, we selected the RBF kernel and tuned the misclassification parameter *C* and the kernel parameter *ϒ* using a two-dimensional grid search where C was chosen from the set {10^−5^, 10^−4^, 10^−3^, …, 10^6^, 10^7^}, and *ϒ* was chosen from the set {10^−8^, 10^−7^, 10^−6^, …, 10^2^, 10^3^}. All experiments were performed with 5-fold cross validation of 6,000 windows randomly sampled in equal quantities from both positive and negative class sets.

### Training sample size, window size, and margin width

Using the optimized SVM-RBF hyperparameters, we tested how window size, training sample size, and margin width affect the performance of this classifier. We tested window size range from 500 bp to 2,000 bp with 500 base pair increments (Additional file 1: Figure S2A). We observed that short windows may not be able to capture enough context around the gene, while long windows resulted in increased computational cost and were problematic for the *P. falciparum* genome, where the average length of intergenic regions is 1,694 bases [4]. For margin width, we tested 25 bp, 50 bp and 100 bp (Additional file 1: Figure S2B). After testing different window sizes and margin widths in cross-validation experiments, we observed that the best recall rate is obtained using a window size of 1,500 bp and a margin width of 50 bp, which were selected as parameters for the final classifier.

In addition, we used cross-validation experiments to test the relationship between training sample size and the performance of the trained classifiers. We ran cross-validation experiments with training sizes of 2,000, 3,000, 6,000, 9,000, 12,000, and 18,000 windows (Additional file 1: Figure S2C). The results indicated that sample size does not have significant impact on the performance of the classifier, as long the sample is sufficiently large. We decided to use a training sample size of 6,000 windows with equal numbers derived from positive and negative classes, which achieves a good trade-off between computational cost and classifier performance. In contrast to this balanced training set, the vast majority of the windows in the genome are expected to be in the negative class. The imbalance in the test set should be reflected in the training set if the objective was to maximize the convex combination of precision and recall with the same weight. However, instead of optimizing precision, the main purpose of this study is to maximize the recall equally well for both positive and negative classes. The use of an imbalanced training set resulted in little change in recall, and we therefore used a balanced training set for this study. With these optimized parameters, we obtained average total recall rates of 0.94 for gene start classifiers trained on B1 data set, 0.92 for gene start classifiers trained on B2 data set, 0.94 for gene end classifiers trained on B1 data set and 0.93 for gene end classifiers trained on B2 data set (Additional file 1: Table S1). The averaged total recall rate was 0.93 for all classifiers. The default confidence probability cutoff value for SVM classifier used here is 0.5. To increase the confidence of our gene prediction method, we tested different confidence probability cutoff values (0.6, 0.7, 0.8, 0.9) and observed that the number of predicted genes decreases as the cutoff value increases. We found that cutoff value of 0.7 gave the best trade-off between a reasonable number of predicted genes and a sufficiently high confidence in their prediction for both data sets B1 and B2.

### Reverse transcription polymerase chain reaction (RT-PCR)

Twenty-one highly confident gene candidates that were predicted using both data set B1 and B2 were selected based on different combinations of RNA-Seq and polysomal RNA-Seq expression profiles (i.e. 3 genes showing high signals in both RNA-Seq and Poly-seq, 6 genes showing a high signal in only one of the profiles, and 12 genes showing low signals in both profiles). Total RNA was isolated from 10 ml of non-synchronous erythrocytic stage *P. falciparum* culture. To remove genomic DNA contamination, RNA samples were treated twice with 4 U DNase I (Life Technologies) per 10 μg of RNA for 30 min at 37 °C. DNase I was inactivated by the addition of EDTA to a final concentration of 1 mM. DNase-treated total RNA was then mixed with 0.1 μg of random hexamers, 0.6 μg of oligo-dT(20), and 2 μl 10 mM dNTP mix (Life Technologies) in total volume of 10 μl, incubated for 10 min at 70 °C and then chilled on ice for 5 min. This mixture was added to a solution containing 4 μl 10X RT buffer, 8 μl 20 mM MgCl_2_, 4 μl 0.1 M DTT, 2 μl 20 U/μl SuperaseIn and 1 μl 200 U/μl SuperScript III Reverse Transcriptase (all from Life Technologies). First-strand cDNA was synthesized by incubating the sample for 10 min at 25 °C, 50 min at 50 °C, and finally 5 min at 85 °C. The absence of genomic DNA contamination was validated using a primer set targeting an intergenic region and a primer set targeting PfAlba3 (PF3D7_1006200) from inside exon 1 to within exon 2. Amplification of genomic DNA should give a product with a size of 429 bp including the intronic sequence, whereas amplification of cDNA should result in a fragment with a size of 164 bp. All 21 PCRs testing transcription activity of predicted genes were performed using 3 μl of the first-strand cDNA mixture with approximately 10 pmole of both forward and reverse primers. DNA was incubated for 5 min at 95 °C, then 30 s at 98 °C, 30 s at 55 °C, 30 s at 62 °C for 35 cycles. 5 μl of each PCR sample was used for agarose gel electrophoresis. For each primer set, PCR efficiency was tested using genomic DNA under the same amplification conditions as described above. All primer used for PCR validation are listed in Additional file 3.

### Coverage plots and histone variant analysis

Sequence reads for ChIP-Seq experiments of *P. falciparum* nucleosome variant H2A.Z [30] (SRP003508) and histone marks H3K36me3 and H3K4me3 [37] (SRP022761) were downloaded and mapped to *P. falciparum* 3D7 genome version 9 using bowtie with default error rates. Coverage profiles for each time point were then generated using BEDtools [32]. For each histone variant, coverage profiles from different time points were summed to generate a combined profile. Sequence coverage for regions 750 bp before and after start and end codons of regions of interest were extracted from the summed coverage profiles. Averaged values for each relative position were then calculated and used to generate coverage plots using R.

### Ethics statement

No ethics approval was required for this study.

### Availability of supporting data

All the supporting data are included as additional files.

## Additional files

Additional file 1: Figure S1. Supervised machine learning approach for novel gene detection. **Figure S2.** Optimization of classifier parameters. **Table S1.** Classifier performance and measurements. **Figure S3.** Density plots of various characteristics of predicted gene regions versus intergenic regions and annotated coding and non-coding genes in *P. falciparum*. **Figure S4.** Coverage profile of histone variants around gene boundaries. (PDF 1149 kb) Additional file 2:
**List of predicted gene regions and their characteristics.** (XLSX 79 kb) Additional file 3:
**Primes used for predicted gene validation.** (XLSX 12 kb)

## Abbreviations

EST: expressed sequence tag
lncRNA: long non-coding RNA
ORF: open reading frame
PCA: principal component analysis
RBF: radial basis function
refseq_rna: reference RNA sequence
RT-PCR: reverse transcription polymerase chain reaction
SVM: support vector machine
TERRA: telomeric repeat-containing lncRNAs
TSS: transcription start sites
