# Supplementary material for: Analysis of nucleosome positioning landscapes enables gene discovery in the human malaria parasite Plasmodium falciparum
Source: BMC Genomics. 2015 Nov 25;16:1005. doi: 10.1186/s12864-015-2214-9 (PMC4658763; doi:10.1186/s12864-015-2214-9)
Supplement: Additional file 1: Figure S1. — Supervised machine learning approach for novel gene detection. Figure S2. Optimization of classifier parameters. Table S1. Classifier performance and measurements. Figure S3. Density plots of various characteristics of predicted gene regions versus intergenic regions and annotated coding and non-coding genes in P. falciparum. Figure S4. Coverage profile of histone variants around gene boundaries. (PDF 1149 kb) [file 12864_2015_2214_MOESM1_ESM.pdf]

Supplementary Figures and Tables to:

Analysis of Nucleosome Positioning Landscapes Enables Gene Discovery in the Human Malaria Parasite *Plasmodium falciparum*

Xueqing Lu[1†], Evelien M. Bunnik[1†], Neeti Pokhriyal[2], Sara Nasseri[2], Stefano Lonardi[2], Karine G. Le Roch[1]*

[1]Department of Cell Biology and Neuroscience, Institute for Integrative Genome Biology, Center for Disease Vector Research, University of California, Riverside, 900 University Avenue, Riverside, CA 92521, USA.

[2]Department of Computer Science and Engineering, University of California, Riverside, 900 University Avenue, Riverside, CA 92521, USA.

† Equal contributors

* Corresponding author. Email: karine.leroch@ucr.edu

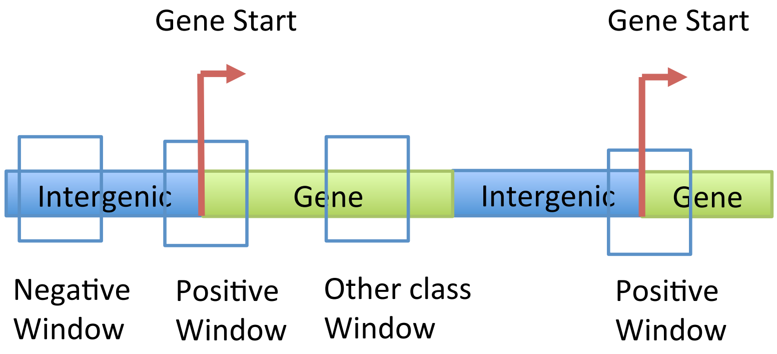

Supplementary Figure 1. Supervised machine learning approach for novel gene detection. Using a sliding window method, the genome-wide nucleosome positioning data set was converted into a set of subsequences ("windows"), where each window is a vector of length w, and each position is a numeric value representing the summed number of mapped reads. A label was then assigned to each of the windows based on the presence of a gene start. A binary classifier for gene start recognition was trained on gene start-containing windows (positive class) and intergenic windows (negative class) with support vector machine (SVM), RBF kernel. A similar approach was used to train a classifier for the detection of gene ends.

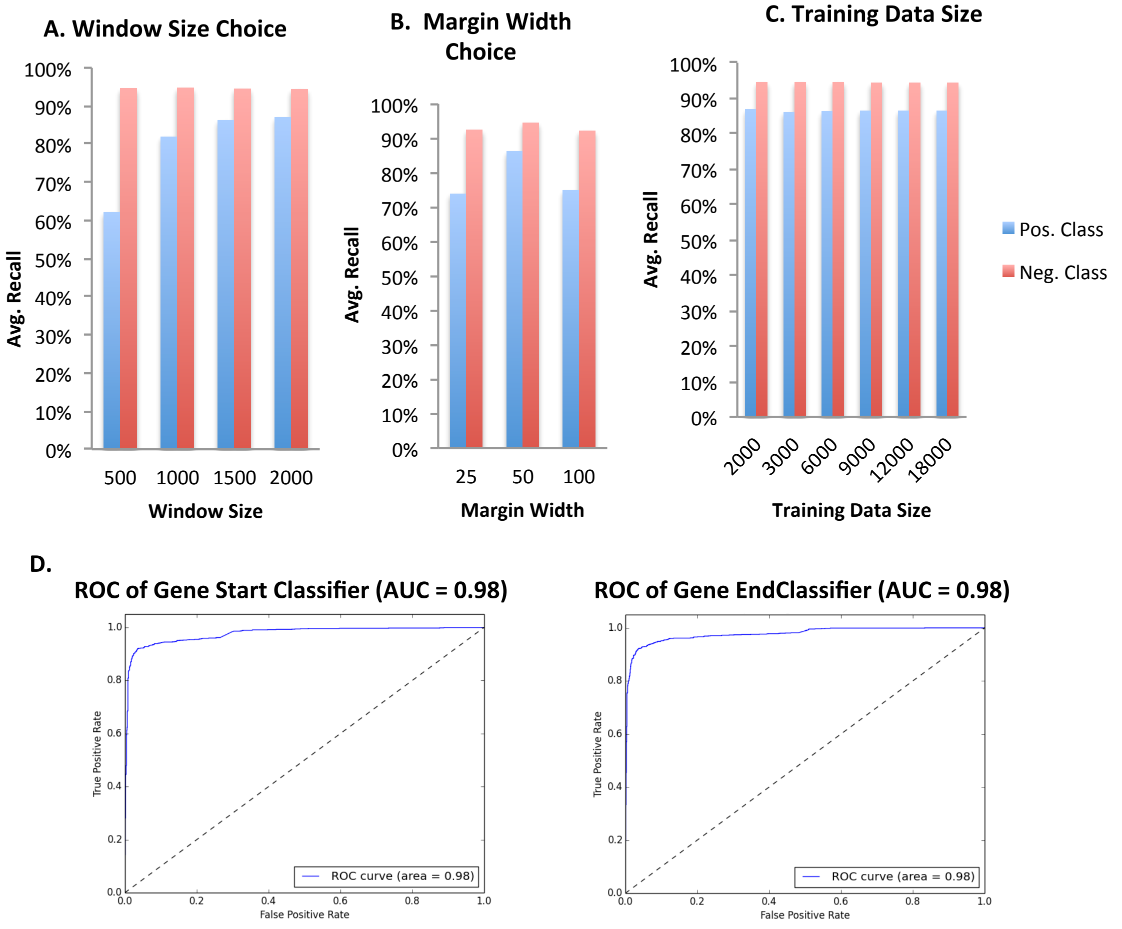

Supplementary Figure 2. Optimization of classifier parameters trained on the positive strand of data set B1. Average recall rates for gene start detection from 10 cross-validation experiments for window size **(A)**, margin width **(B)**, and training data size **(C)**. After comparing the recall rate for each parameter, the optimized classifier was trained using 6,000 windows of 1,500 bp with 50 bp margin width drawn in equal quantities from both positive and negative class. The ROC curves for optimized gene start and gene end classifiers are reported in (**D**). Results of optimization experiments for classifiers trained on the negative strand of data set B1 and classifiers trained on data set B2 were very similar and are therefore not shown. A detailed explanation of classifier optimization is presented in the Material and Methods section.

B1 dataset							
**Gene Start**				**Gene End**			
	**Class**	**Recall**	**AUC**		**Class**	**Recall**	**AUC**
**Positive Strand Classifier**	Intergenic (0)	0.97	0.98	**Positive Strand Classifier**	Intergenic (0)	0.94	0.98
	Gene(1)	0.91			Gene(1)	0.92	
	total	0.94			total	0.93	
	**Class**	**Recall**	**AUC**		**Class**	**Recall**	**AUC**
**Negative Strand Classifier**	Intergenic (0)	0.94	0.98	**Negative Strand Classifier**	Intergenic (0)	0.96	0.98
	Gene(1)	0.94			Gene(1)	0.94	
	total	0.94			total	0.95	

B2 dataset							
**Gene Start**				**Gene End**			
	**Class**	**Recall**	**AUC**		**Class**	**Recall**	**AUC**
**Positive Strand Classifier**	Intergenic (0)	0.92	0.96	**Positive Strand Classifier**	Intergenic (0)	0.92	0.97
	Gene(1)	0.90			Gene(1)	0.92	
	total	0.91			total	0.92	
	**Class**	**Recall**	**AUC**		**Class**	**Recall**	**AUC**
**Negative Strand Classifier**	Intergenic (0)	0.92	0.97	**Negative Strand Classifier**	Intergenic (0)	0.93	0.98
	Gene(1)	0.93			Gene(1)	0.94	
	total	0.92			total	0.93	

Supplementary Table 1: Classifier performance records.

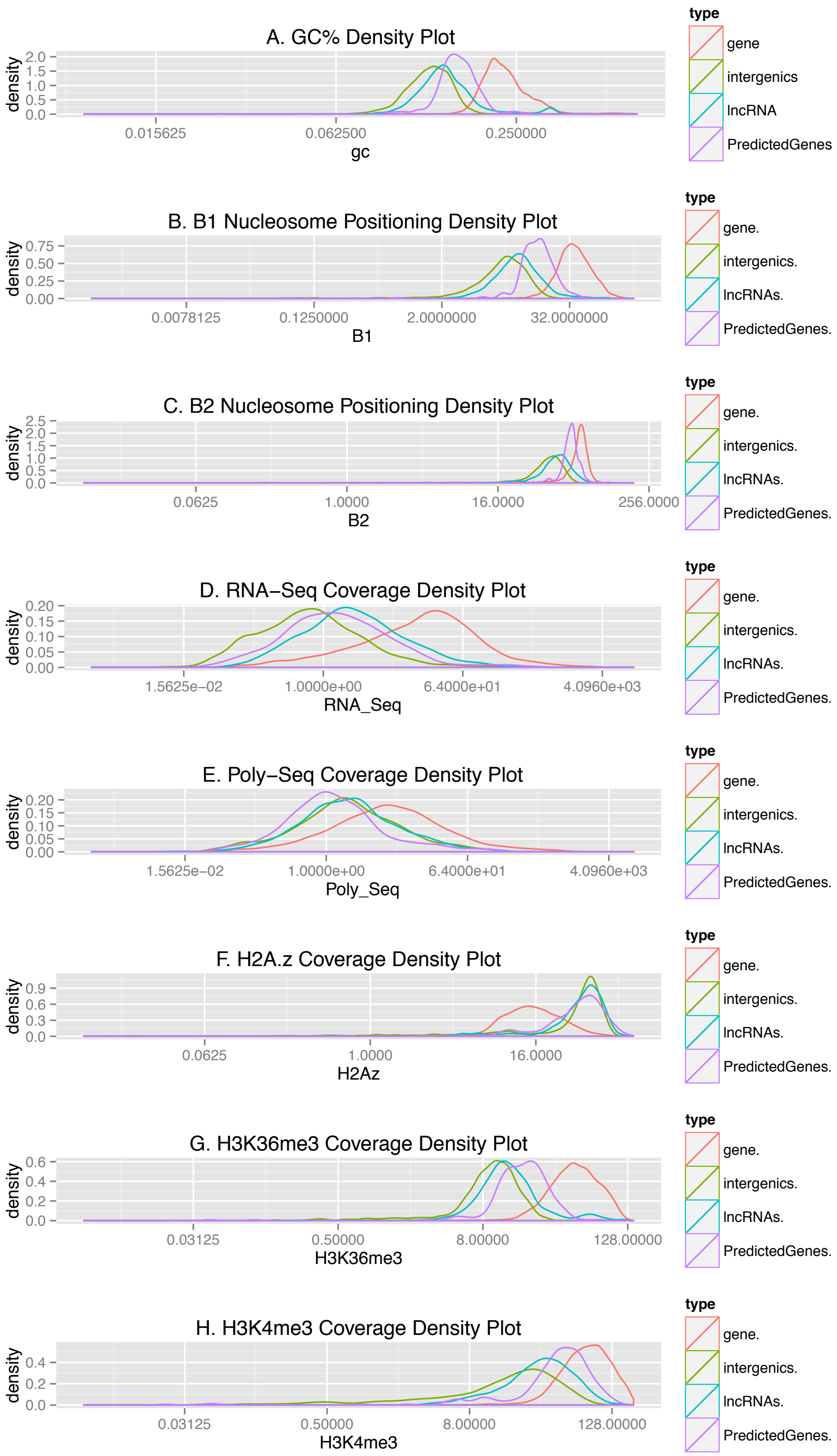

Supplementary Figure 3. Density plots of various characteristics of predicted gene regions versus intergenic regions and annotated coding and non-coding genes in *P. falciparum*.

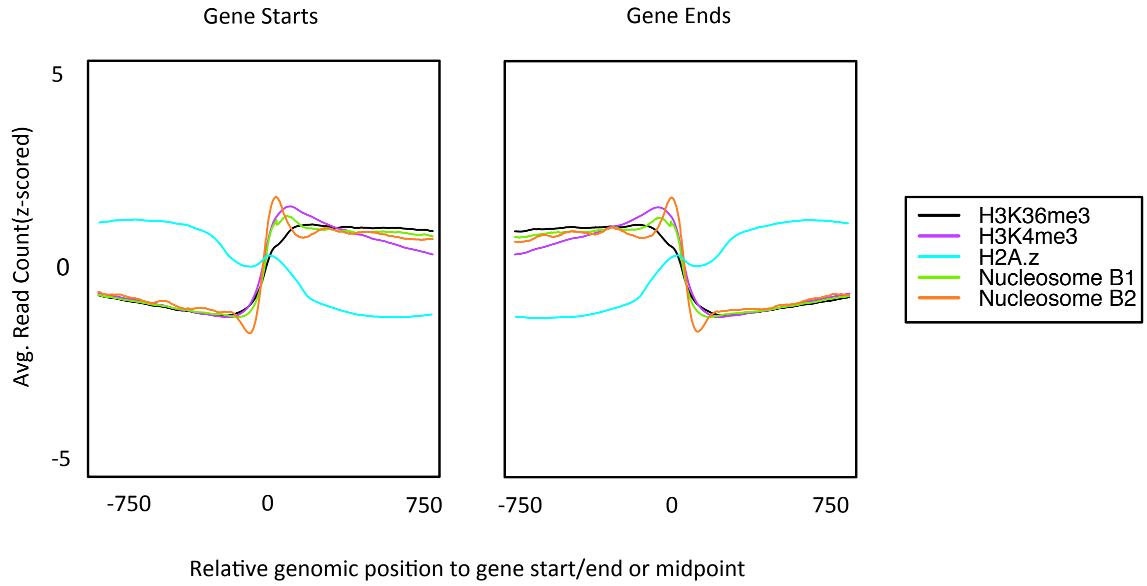

Supplementary Figure 4: Coverage profiles of histone variants around gene boundaries.
